# Ukrainian migrants’ and war refugees’ admissions to hospital: evidence from the Polish Nationwide General Hospital Morbidity Study, 2014–2022

**DOI:** 10.1186/s12889-023-17202-5

**Published:** 2023-11-24

**Authors:** Katarzyna Lewtak, Anna Poznańska, Krzysztof Kanecki, Piotr Tyszko, Paweł Goryński, Krzysztof Jankowski, Aneta Nitsch-Osuch

**Affiliations:** 1 Department of Health Promotion and Prevention of Chronic Diseases, National Institute of Public Health NIH – National Research Institute, 24 Chocimska Street, 00-791 Warsaw, Poland; 2 Department of Social Medicine and Public Health, Medical University of Warsaw, Warsaw, Poland; 3 Department of Population Health Monitoring and Analysis, National Institute of Public Health NIH – National Research Institute, Warsaw, Poland; 4 Institute of Rural Health in Lublin, Lublin, Poland; 5 National Geriatrics, Rheumatology and Rehabilitation Institute, Warsaw, Poland

**Keywords:** Migrant health, Refugee health, Hospital admissions, Hospital morbidity, Ukraine, Poland

## Abstract

**Background:**

Considering the rapid influx of Ukrainian migrants and war refugees into Poland, the knowledge of their health condition is becoming increasingly important for health system policy and planning.

The aim of the study was to assess war-related changes in the frequency and structure of hospitalizations among Ukrainian migrants and refugees in Poland.

**Methods:**

The study is based on the analysis of hospital admission records of Ukrainian patients, which were collected in the Nationwide General Hospital Morbidity Study from 01.01.2014 to 31.12.2022.

**Results:**

In the study period, 13,024 Ukrainians were hospitalized in Poland, 51.7% of whom had been admitted to hospital after February 24, 2022. After the war broke out, the average daily hospital admissions augmented from 2.1 to 21.6 person/day. A noticeable increase in the share of women (from 50% to 62%) and children (from 14% to 51%) was also observed. The average age of patients fell from 33.6 ± 0.2 years to 24.6 ± 0.3 years. The most frequently reported hospital events among the migrants until 23.02.2022 were injuries (S00-T98) – 26.1%, pregnancy, childbirth and the puerperium (O00-O99) – 18.4%, and factors influencing health status and contact with health services (Z00-Z99) – 8.4%. After the war started, the incidence of health problems among migrants and war refugees changed, with pregnancy, childbirth and the puerperium (O00-O99) being the most common – 14.9%, followed by abnormal clinical and lab findings (R00-R99) – 11.9%, and infectious and parasitic diseases (A00-B99) – 11.0%.

**Conclusions:**

Our findings may support health policy planning and delivering adequate healthcare in refugee-hosting countries.

## Background

Since the Russian invasion of Ukraine started on February 24, 2022 over 12 million people have crossed the Polish-Ukrainian border, including 1,602,062 refugees registered in Poland with temporary protection status (as of 16.05.2023) [1, 2].

An increased influx of Ukrainian citizens into Poland has been reported since 2014, as a result of the political and economic situation in that country [3]. Refugee crisis has an impact on the refugee hosting country with regard to its healthcare, economy, political and social life. It is a huge challenge for the public health system to provide refugees with appropriate healthcare [4–8]. World Health Organization (WHO) stresses the need to implement practical action towards universal healthcare that leaves no one invisible, no one without essential and quality health services, no one behind [9].

Two days after the outbreak of the war, on February 26th, 2022, Polish government declared that each person from Ukraine who had crossed the Polish border since the onset of war would have access to free medical services at all levels (i.e. visits to family doctors, specialized outpatient care, inpatient treatment, psychiatric treatment, rehabilitation (except health resorts) and dental services) under the same rules and in the same scope as persons covered by obligatory or voluntary health insurance in Poland.

Studies on the health condition of migrants and war refugees and the use of health services by them are of key importance for health policy and strategic planning of healthcare in the host countries [10–14].

Hospital care is an important element of health system and hospital admissions are often used to describe the level of bad health condition—diseases and health problems among migrants and war refugees [15–18].

The aim of the study was to assess changes in the frequency of hospitalized morbidity, its structure, and in-hospital fatality in migrants and refugees hospitalized in Poland after the outbreak of the Russian-Ukrainian war.

## Methods

### Study design and data source

Data on the hospitalization of Ukrainian citizens in Polish hospitals come from the Nationwide General Hospital Morbidity Study (NGHMS), conducted by the National Institute of Public Health NIH – National Research Institute within the framework of the Statistical Survey Programme of Official Statistics. All hospitals, except for psychiatric facilities, are legally bound to participate in this study [19].

For each of hospitalized patient, we obtained the following data: age, gender, admission and discharge dates, discharge status (including death and causes of death), as well as main discharge diagnosis. The analysis was based on the data collected from 1.01.2014 to 31.12.2022. Two periods were analysed: before (01.01.2014–23.02.2022) and after (24.02.2022–31.12.2022) the outbreak of the war.

For our analysis, hospital admissions of Ukrainian citizens, including paediatric patients (aged < 18 years) and adults (≥ 18 years), were categorized by their principal discharge diagnostic codes (the main condition responsible for the stay in hospital), according to the 10th revision of the International Classification of Diseases (ICD-10).

Twenty admission categories were examined, according to the chapter blocks of the ICD-10.

In the study, the frequency and causes of the in-hospital fatality were also determined.

### Statistical analysis

The distribution of the causes of hospitalization in the compared periods (for the total number of patients and for two distinguished age categories: adults; and children and adolescents under 18 years of age) is presented in absolute numbers and percentages with the 95% confidence intervals. The change in the share of a given cause of hospitalization is presented as a multiple (in tables) or as a difference expressed in percentage points. The statistical significance of differences in the prevalence of particular causes of hospitalization was assessed using the two-tailed Pearson's χ^2^ test or Fisher exact test. The significance level for all tests was assumed to amount to 0.05.

The mean age in the analysed subgroups is presented as the mean value ± mean standard error and as the median. The calculations were executed with the SPSS version 21.

## Results

### Baseline data review

According to the NGHMS, 13,024 Ukrainian citizens were admitted to Polish hospitals (except for psychiatric facilities) in the analysed period, including 5,676 men and 7,348 women. 6,728 persons (51.7%) were admitted to hospital after the outbreak of war (24.02.2022), including 2534 men and 4194 women (Table 1).

**Table 1:** General characteristics of Ukrainian war refugees and migrants admitted to Polish hospitals between 01.01.2014 and 31.12.2022

Variables	01.01.2014-23.02.2022		24.02.2022-31.12.2022		Total	
	n	%	n	%	n	%
Gender						
Male	3142	49.9%	2534	37.7%	5676	43.6%
Female	3154	50.1%	4194	62.3%	7348	56.4%
Total	6296	100.0%	6728	100.0%	13024	100.0%
Age						
0–17 years	857	13.6%	3411	50.7%	4268	32.8%
≥ 18 years	5418	86.1%	3316	49.3%	8734	67.1%
Missing records	21	0.3%	1	0.0%	22	0.2%
Total	6296	100.0%	6728	100.0%	13024	100.0%
Minors (0–17 years)						
Male	536	62.5%	1862	54.6%	2398	56.2%
Female	321	37.5%	1549	45.4%	1870	43.8%
Total	857	100.0%	3411	100.0%	4268	100.0%
Adults (≥ 18 years)						
Male	2590	47.8%	672	20.3%	3262	37.3%
Female	2828	52.2%	2644	79.7%	5472	62.7%
Total	5418	100.0%	3316	100.0%	8734	100.0%

**Fig. 1:**
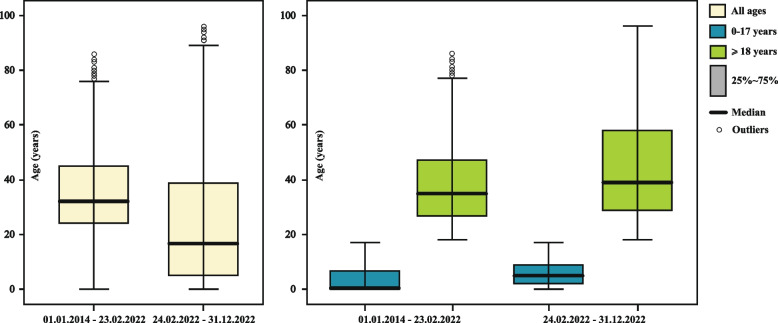
Age distribution of the hospitalized Ukrainian citizens by period of hospital admission and age groups (in box plots)

The mean age of patients in the entire analysed period was 28.9 ± 0.2, and the median age was 28 years. After 24.02.2022, the age structure of patients changed (Fig. 1). The mean age of the hospitalized persons decreased from 33.6 ± 0.2 years to 24.6 ± 0.3 years, the median fell from 32 to 17 years as a result of the increase in the number of young patients due to the influx of a large number of children to Poland. Before the outbreak of the war, the majority (50.4%) of hospitalized patients under 18 were newborns, currently their share has decreased to 10.2%. The average age of hospitalized children (0–17 years) increased from 4.2 ± 0.2 years to 5.9 ± 0.1 years; the median increased from 0 to 5 years. The average age of hospitalized adults increased from 38.2 ± 0.2 years to 43.9 ± 0.3 years, whereas the median increased from 35 to 39 years.

Before the war, the share of men and women was almost the same (49.9% vs 50.1%), with boys clearly dominating among paediatric patients (62.5%), and women being slightly more predominant among adults (52.2%) (Table 1). After 24.02.2022, an increase in female patients was observed (62.3%). Women constituted up to 79.7% of the hospitalized adults, while the sex disproportion among children decreased (the share of boys was 54.6%). The changes in the gender distribution were statistically significant, both in the group of children (*p* < 0.001) and adults (*p* < 0.001).

### Frequency and structure of hospitalizations

Before the outbreak of the war, the average of 2.1 person/day were admitted to hospital, and after 24.02.2022 this number increased to 21.6 patients a day. Considering the subgroup of children, before the war 0.3 child was admitted to hospital per day, and after the outbreak of the war there were 11.0 child hospital admissions/day. Regarding adult patients, 1.8 persons and 10.7 persons were admitted to hospital before and after 24.02.2022, respectively. Before 24.02.2022 the most common causes of hospitalization among Ukrainian citizens in Poland were injuries and poisoning (ICD-10: S00-T98), which concerned over a quarter of all patients (26.1 [25.1- 27.2]%) (Table 2).

**Table 2:** Hospitalization events by ICD-10 chapter among Ukrainian war refugees and migrants (in total) in the period before (01.01.2014–23.02.2022) and after (24.02.2022–31.12.2022) the outbreak of the war

Group of diagnoses (ICD-10 chapters)	01.01.2014-23.02.2022			24.02.2022-31.12.2022			Percentage change (multiple)	p
	**rank**	**n**	**% [95% CI]**	**rank**	**n**	**% [95% CI]**		
Injury, poisoning and certain other consequences of external causes (S00-T98)	1	1646	26.1 [25.1–27.2]	4	628	9.3 [8.6–10.0]	**↓ 2.8**	** < 0.001**
Pregnancy, childbirth and the puerperium (O00-O99)	2	1159	18.4 [17.5–19.4]	1	1005	14.9 [14.1–15.8]	**↓ 1.2**	** < 0.001**
Factors influencing health status and contact with health services (Z00-Z99)	3	530	8.4 [7.7–9.1]	6	495	7.4 [6.7–8.0]	**↓ 1.1**	**0.025**
Neoplasms (C00-D48)	4	395	6.3 [5.7–6.9]	5	539	8.0 [7.4–8.7]	**↑ 1.3**	** < 0.001**
Symptoms, signs and abnormal clinical and laboratory findings, not elsewhere classified (R00-R99)	5	381	6.1 [5.5–6.6]	2	798	11.9 [11.1–12.6]	**↑ 2.0**	** < 0.001**
Diseases of the genitourinary system (N00-N99)	6	376	6.0 [5.4–6.6]	8	329	4.9 [4.4–5.4]	**↓ 1.2**	**0.006**
Diseases of the circulatory system (I00-I99)	7	362	5.7 [5.2–6.3]	10	275	4.1 [3,6–4.6]	**↓ 1.4**	** < 0.001**
Diseases of the digestive system (K00-K93)	8	289	4.6 [4.1–5.1]	9	276	4.1 [3,6–4.6]	**↓** 1.1	NS
Diseases of the musculoskeletal system and connective tissue (M00-M99)	9	200	3.2 [2.7–3.6]	18	86	1.3 [1.0–1.5]	**↓ 2.5**	** < 0.001**
Certain conditions originating in the perinatal period (P00-P96)	10	160	2.5 [2.2–2.9]	14	123	1.8 [1.5–2.1]	**↓ 1.4**	**0.005**
Diseases of the respiratory system (J00-J99)	11	145	2.3 [1.9–2.7]	7	464	6.9 [6.3–7.5]	**↑ 3.0**	** < 0.001**
Diseases of the nervous system (G00-G99)	12	141	2.2 [1.9–2.6]	17	112	1.7 [1.4–2.0]	**↓ 1.3**	**0.018**
Diseases of the skin and subcutaneous tissue (L00-L99)	13	103	1.6 [1.3–1.9]	16	113	1.7 [1.4–2.0]	↑1.0	NS
Diseases of the blood and blood-forming organs and certain disorders involving the immune mechanism (D50-D89)	14	101	1.6 [1.3–1.9]	15	120	1.8 [1.5–2.1]	**↑** 1.1	NS
Endocrine, nutritional and metabolic diseases (E00-E90)	15	83	1.3 [1.0–1.6]	11	242	3.6 [3.2–4.0]	**↑ 2.7**	** < 0.001**
Certain infectious and parasitic diseases (A00-B99)	16	61	1.0 [0.7–1.2]	3	740	11.0 [10.3–11.7]	**↑ 11.4**	** < 0.001**
Congenital malformations, deformations and chromosomal abnormalities (Q00-Q99)	17	50	0.8 [0.6–1.0]	20	43	0.6 [0.4–0.8]	↓ 1.2	NS
Diseases of the ear and mastoid process (H60-H95)	18	45	0.7 [0.5–0.9]	19	59	0.9 [0.7–1.1]	**↑** 1.2	NS
Codes for special purposes (U00-U85)	19	44	0.7 [0.5–0.9]	12	149	2.2 [1.9–2.6]	**↑ 3.2**	** < 0.001**
Diseases of the eye and adnexa (H00-H59)	20	25	0.4 [0.2–0.6]	13	132	2.0 [1.6–2.3]	**↑ 4.9**	** < 0.001**
**TOTAL**		**6296**	**100**		**6728**	**100**		

For diagnoses whose share changed in a statistically significant way after 24.02.2022, the multiple of the percentage change and the p-value are bolded

*Abbreviations*: *NS* not significant. *95% CI* 95% confidence interval

Second most frequently reported health problems were related to pregnancy, childbirth and the puerperium (O00-O99), which concerned 18.4 [17.5–19.4]% of patients; and they were followed by the causes defined as factors influencing the state of health (Z00-Z99), which concerned 8.4 [7.7–9.1]% patients, including liveborn infants (Z38), which amounted 45.4% of hospitalizations in this group of causes and 3.9 [3.4–4.4]% of all hospitalization cases; as well as chemotherapy (Z51.1), which concerned 34.0% in this group of causes and 2.9 [2.5–3.4]% of all hospitalization cases. Neoplasms (C00-D48) were the fourth most frequent cause of all hospitalizations (6.3 [5.7–6.9]%).

After February 24, 2022, the structure of the causes of hospitalization changed significantly (Table 2). Over 1,000 patients (14.9 [14.1–15.8]%) were hospitalized for pregnancy, childbirth and the puerperium (O00-O99). Symptoms, signs and abnormal clinical and laboratory findings (R00-R99) were the second most frequently reported causes of hospitalization (11.9 [11.1–12.6]%), the majority of which (60.3%) were related to digestive system symptoms (R10-R19), and fever of other and unknown origin (R50) (12.8%), which were the cause of 7.1 [6.5–7.8]% and 1.5 [1.2–1.8]% of all hospitalizations, respectively. The third most significant cause of hospitalizations were infectious and parasitic diseases (A00-B99) (11.0 [10.3–11.7]%), and the fourth one—injury, poisoning and certain other consequences of external causes (S00-T98) (9.3 [8.6–10.0]%).

The observed change in the distribution of the causes of hospitalization is primarily a consequence of changes in the age of the patients. Before February 24, 2022, the vast majority (86.3%) of Ukrainian citizens hospitalizations in Poland concerned adults. Problems characteristic of this age group (Table 3), i.e., injuries (S00-T98), causes related to motherhood (O00-O99) and neoplasms (C00-D48), dominated among all patients.

**Table 3:** Hospitalization events by ICD-10 chapter among Ukrainian war refugees and migrants (adults aged 18 and more) in the period before (01.01.2014 – 23.02.2022) and after (24.02.2022–31.12.2022) the war outbreak

Group of diagnoses (ICD-10 chapters)	01.01.2014-23.02.2022			24.02.2022-31.12.2022			Percentage change (multiple)	p
	**rank**	**n**	**% [95% CI]**	**rank**	**n**	**% [95% CI]**		
Injury, poisoning and certain other consequences of external causes (S00-T98)	1	1576	29.1 [27.9–30.3]	3	297	9.0 [8.0–9.9]	**↓ 3.2**	** < 0.001**
Pregnancy, childbirth and the puerperium (O00-O99)	2	1159	21.4 [20.3–22.5]	1	994	30.0 [28.4–31.5]	**↑ 1.4**	** < 0.001**
Neoplasms (C00-D48)	3	360	6.6 [6.0–7.3]	2	376	11.3 [10.3–12.4]	**↑ 1.7**	** < 0.001**
Diseases of the circulatory system (I00-I99)	4	357	6.6 [5.9–7.2]	6	235	7.1 [6.2–8.0]	↑ 1.1	NS
Symptoms, signs and abnormal clinical and laboratory findings, not elsewhere classified (R00-R99)	5	352	6.5 [5.8–7.2]	4	270	8.1 [7.2–9.1]	**↑ 1.3**	**0.004**
Diseases of the genitourinary system (N00-N99)	6	349	6.4 [5.8–7.1]	5	267	8.1 [7.2–9.0]	**↑ 1.3**	**0.004**
Diseases of the digestive system (K00-K93)	7	272	5.0 [4.4–5.6]	8	148	4.5 [3.8–5.2]	↓ 1.1	NS
Factors influencing health status and contact with health services (Z00-Z99)	8	263	4.9 [4.3–5.4]	7	228	6.9 [6.0–7.7]	**↑ 1.4**	** < 0.001**
Diseases of the musculoskeletal system and connective tissue (M00-M99)	9	183	3.4 [2.9–3.9]	13	54	1.6 [1.2–2.1]	**↓ 2.1**	** < 0.001**
Diseases of the nervous system (G00-G99)	10	118	2.2 [1.8–2.6]	15	49	1.5 [1.1–1.9]	**↓ 1.5**	**0.020**
Diseases of the respiratory system (J00-J99)	11	111	2.0 [1.7–2.4]	11	57	1.7 [1.3–2.2]	↓ 1.2	NS
Diseases of the skin and subcutaneous tissue (L00-L99)	12	97	1.8 [1.4–2.1]	12	56	1.7 [1.3–2.1]	↓ 1.1	NS
Endocrine, nutritional and metabolic diseases (E00-E90)	13	47	0.9 [0.6–1.1]	10	58	1.7 [1.3–2.2]	**↑ 2.0**	** < 0.001**
Codes for special purposes (U00-U85)	14	43	0.8 [0.6–1.0]	14	52	1.6 [1.1–2.0]	**↑ 2.0**	** < 0.001**
Diseases of the ear and mastoid process (H60-H95)	15	37	0.7 [0.5–0.9]	18	15	0.5 [0.2–0.7]	↓ 1.5	NS
Certain infectious and parasitic diseases (A00-B99)	16	30	0.6 [0.4–0.8]	17	32	1.0 [0.6–1.3]	**↑ 1.7**	**0.026**
Diseases of the blood and blood-forming organs and certain disorders involving the immune mechanism (D50-D89)	17	29	0.5 [0.3–0.7]	16	47	1.4 [1.0–1.8]	**↑ 2.6**	** < 0.001**
Diseases of the eye and adnexa (H00-H59)	18	20	0.4 [0.2–0.5]	9	79	2.4 [1.9–2.9]	**↑ 6.5**	** < 0.001**
Congenital malformations, deformations and chromosomal abnormalities (Q00-Q99)	19	15	0.3 [0.1–0.4]	2	19	0.1 [0.0–0.1]	**↓ 4.6**	**0.026**
Certain conditions originating in the perinatal period (P00-P96)	N/A	N/A	N/A	N/A	N/A	N/A	N/A	N/A
**TOTAL**		**5418**	**100**		**3316**	**100**		

Missing 22 records on patient’s age (21 cases before the war outbreak and one case after February 24, 2022) in the database

For diagnoses whose share changed in a statistically significant way after 24.02.2022, the multiple of the percentage change and the p-value are bolded

*Abbreviations*: *NS* not significant, *N/A* not applicable, *95% CI* 95% confidence interval

After the outbreak of the war 50.7% of the hospitalized were patients under 18 years of age (13.7% before the outbreak of the war). The causes prevalent among children are dominant among all patients, and these are infectious and parasitic diseases (A00-B99) (mainly viral and other specified intestinal infections (A08), other gastroenteritis and colitis of infectious and unspecified origin (A09)), symptoms, signs and abnormal clinical and laboratory findings (R00-R99) (mainly digestive system disorders (60.0%) and fever of unknown origin (18.0%)), and diseases of the respiratory system (J00-J99) (Table 4).

**Table 4:** Hospitalization events by ICD-10 chapter among Ukrainian war refugees and migrants (children and adolescents under 18 years of age) admitted to hospitals in Poland in the period before (01.01.2014–23.02.2022) and after (24.02.2022–31.12.2022) the war outbreak

Group of diagnoses (ICD-10 chapters)	01.01.2014-23.02.2022			24.02.2022-31.12.2022			Percentage change (multiple)	p
	**rank**	**n**	**% [95% CI]**	**rank**	**n**	**% [95% CI]**		
Factors influencing health status and contact with health services (Z00-Z99)	1	267	31.2 [28.1–34.3]	5	267	7.8 [6.9–8.7]	**↓ 4.0**	** < 0.001**
Certain conditions originating in the perinatal period (P00-P96)	2	160	18.7 [16.1–21.3]	9	123	3.6 [3.0–4.2]	**↓ 5.2**	** < 0.001**
Diseases of the blood and blood-forming organs and certain disorders involving the immune mechanism (D50-D89)	3	72	8.4 [6.5–10.3]	11	73	2.1 [1.7–2.6]	**↓ 3.9**	** < 0.001**
Injury, poisoning and certain other consequences of external causes (S00-T98)	4	57	6.7 [5.0–8.3]	4	331	9.7 [8.7–10.7]	**↑ 1.5**	**0.005**
Endocrine, nutritional and metabolic diseases (E00-E90)	5	36	4.2 [2.9–5.5]	6	184	5.4 [4.6–6.2]	**↑** 1.3	NS
Congenital malformations, deformations and chromosomal abnormalities (Q00-Q99)	6	35	4.1 [2.8–5.4]	17	41	1.2 [0.8–1.6]	**↓ 3.4**	** < 0.001**
Neoplasms (C00-D48)	7–8	34	4.0 [2.7–5.3]	7	162	4.7 [4.0–5.5]	↑ 1.2	NS
Diseases of the respiratory system (J00-J99)	7–8	34	4.0 [2.7–5.3]	3	407	11.9 [10.8–13.0]	**↑ 3.0**	** < 0.001**
Certain infectious and parasitic diseases (A00-B99)	9	31	3.6 [2.4–4.9]	1	708	20.8 [19.4–22.1]	**↑ 5.7**	** < 0.001**
Symptoms, signs and abnormal clinical and laboratory findings, not elsewhere classified (R00-R99)	10	29	3.4 [2.2–4.6]	2	528	15.5 [14.3–16.7]	**↑ 4.6**	** < 0.001**
Diseases of the genitourinary system (N00-N99)	11	25	2.9 [1.8–4.0]	13	62	1.8 [1.4–2.3]	**↓ 1.6**	**0.042**
Diseases of the nervous system (G00-G99)	12	23	2.7 [1.6–3.8]	12	63	1.8 [1.4–2.3]	↓ 1.5	NS
Diseases of the digestive system (K00-K93)	13	17	2.0 [1.1–2.9]	8	128	3.8 [3.1–4.4]	**↑ 1.9**	**0.011**
Diseases of the musculoskeletal system and connective tissue (M00-M99)	14	14	1.6 [0.8–2.5]	19	32	0.9 [0.6–1.3]	↓ 1.7	NS
Diseases of the ear and mastoid process (H60-H95)	15	8	0.9 [0.3–1.6]	16	44	1.3 [0.9–1.7]	**↑** 1.4	NS
Diseases of the skin and subcutaneous tissue (L00-L99)	16	6	0.7 [0.1–1.3]	14	57	1.7 [1.2–2.1]	**↑ 2.4**	**0.035**
Diseases of the eye and adnexa (H00-H59)	17	5	0.6 [0.1–1.1]	15	53	1.6 [1.1–2.0]	**↑ 2.7**	**0.028**
Diseases of the circulatory system (I00-I99)	18	3	0.4 [0.0–0.7]	18	40	1.2 [0.8–1.5]	**↑ 3.3**	**0.031**
Codes for special purposes (U00-U85)	19	1	0.1 [0–0.3]	10	97	2.8 [2.3–3.4]	**↑ 24.4**	** < 0.001**
Pregnancy, childbirth and the puerperium (O00-O99)	20	0	0	20	11	0.3 [0.1–0.5]	N/A	NS
**TOTAL**		**857**	**100**		**3411**	**100**		

Missing 22 records on patient’s age (21 cases before the war outbreak and one case after February 24, 2022) in the database

For diagnoses whose share changed in a statistically significant way after 24.02.2022, the multiple of the percentage change and the *p*-value are bolded

*Abbreviations*: *NS* not significant, *N/A* not applicable, *95% CI* 95% confidence interval

The share of hospitalizations related to maternal or obstetric causes (O00-O99) decreased by 3.5 percentage points (pp) in the group of all patients (*p* < 0.001, Table 2), and by over 11 pp in the group of women (from 36.7 [35.1–38.4]% to 24.0 [22.7–25.3]%; *p* < 0.001; Table 3). Eleven cases of hospitalizations were recorded for female patients under 18 years of age for this group of causes (Table 4), which concerned exclusively adult women in the period before 24.02.2022.

Apart from changes related to the patients’ age structure after the war outbreak, differences in the frequency of hospitalizations related to particular diseases in both age groups were observed. This effect is particularly visible among children and adolescents. In paediatric patients, the prevalence of infectious and parasitic diseases (A00-B99) increased by over 17 pp, which is statistically significant (*p* < 0.001), similar to the group of adult patients (from 0.6% to 1.0%; *p* = 0.026). The share of hospitalizations related to COVID-19 also increased significantly, by almost 3 pp (*p* < 0.001). Since February 24, 2022 (which is after the peak of the pandemic in Poland), 97 children were hospitalized for COVID-19 (before this date only one child was hospitalized). A statistically significant increase in the frequency of hospitalizations for this reason (by almost 1 pp; *p* < 0.001) was also observed in the group of adults.

The prevalence of hospitalizations due to diseases of the respiratory system and diseases of the digestive system increased significantly among children, by almost 8 pp (*p* < 0.001) and 2 pp (*p* = 0.011), respectively. This was not observed in adults. Additionally, in children the share of hospitalizations for symptoms, signs and abnormal clinical and laboratory findings (R00-R99) increased by more than 12 pp, where digestive system disorders and fever of unknown origin were predominant. For adults this effect was slighter (the share of hospitalization due to R00-R99 increased by almost 2 pp) but also statistically significant (*p* = 0.004).

The share of hospitalizations due to neoplasms (C00-D48) increased by almost 5 pp in adults (*p* < 0.001). Before the war outbreak, in an 8-year period 178 cases of adult hospitalizations were recorded due to chemotherapy (Z51.1) (3.3 [2.8–3.8]% of the total number), after its outbreak, in less than a year – 193 (5.8 [5.2–6.4]%). Adult patients hospitalized due to cancer (C00-D48 and Z51.1) are mostly females (72.1% before the war and as many as 85.6% after its outbreak; *p* < 0.001). The share of children hospitalized for neoplasms increased insignificantly (by 0.7 pp). As a result, the share of neoplasms in the total number of hospitalizations increased by almost 2 pp (*p* < 0.001).

The share of hospitalizations due to injuries (S00-T98) decreased by 20.1 pp (*p* < 0.001) in adults, which may be related to the change in the age structure of the Ukrainians in Poland. Before 24.02.2022, patients over 60 years of age accounted for only 8.0% of hospitalized adults, whereas after the outbreak of the war they constituted 22.1%. The change in the age structure may also justify a statistically significant (*p* < 0.001) decrease in the share of musculoskeletal system diseases (M00-M99) that require hospitalization, which are typical for physical workers. An increase in the share of hospitalizations due to injuries was observed in children and adolescents (by 3.0 pp; *p* = 0.005).

### In-hospital fatality

In the analysed period 96 of 13,024 patients (0.7%) died in hospital. These patients were most often hospitalized due to the circulatory system diseases (I00-I99) (45%), neoplasms (C00-D48) (13%) and injuries (S00-T98) (10%). In-hospital fatality among all patients before February 24, 2022, was 0.9%, whereas after the outbreak of the war it was 0.6%; the difference is statistically significant (*p* = 0.017). In both analysed periods, the deceased were most often hospitalized due to the diseases of the circulatory system (45% for both cases). There were 8 children among the deceased. The mortality rate decreased in this group, it amounted to 0.6% and 0.1% for the two periods, respectively—difference is statistically significant (*p* = 0.011). After the outbreak of the war, in-hospital fatality in adults insignificantly increased from 1.0% to 1.1%.

## Discussion

Information on the causes and frequency of hospitalizations constitutes one of the most important elements in the analysis and assessment of the health condition of the population. Along with other regularly collected health data, this information is important for health policy activities planning.

The health of migrants and refugees is influenced by a number of factors, such as the conditions in their country of origin, why and when they migrated, and their experiences after arrival to the host country. Many studies have confirmed that migrants arrive in the destination country in better health condition than the population of the host country. This phenomenon was called the "healthy immigrant effect" (HIE) [20, 21]. However, HIE is not uniform for all migrants on arrival [20]. This effect is absent in the case of war refugees who are faced with many challenges of fleeing a war-torn country [6, 21]. The use of healthcare services by migrants in the country of destination varies [22]. Hjern et al. showed that higher health services utilization was specifically associated with organized violence in the country of origin [23]. Our study showed a noticeable increase in the daily number of hospital admissions of Ukrainian citizens after the outbreak of the war. Considering the total number of patients, it increased more than tenfold, 5.9 times in adults, and 36.7 times in patients under 18 years of age. This situation brings about many challenges, including organizational ones. Regarding the youngest group of patients, this observation remains in line with the fact that one month of war in Ukraine led to the displacement of 4.3 million children (including 1.8 million children who arrived in the neighboring countries as war refugees), which is more than half of the country's child population [24].

The analysis of main causes of hospitalizations of Ukrainian citizens after February, 24 2022, shows a noticeable increase in the share of chronic diseases demanding specialist treatment, among others, neoplasms (C00-D48), diseases of the respiratory system (J00-J99) and endocrine, nutritional and metabolic diseases (E00-E90), as well as diseases that demanded isolation—infectious diseases (A00-B99) and COVID-19.

The most important hospital discharge diagnoses of adult Ukrainians after February 24, 2022, did not change. The top three causes of hospital admissions remain the same. Only the order of the frequency of occurrence changed. There is a significant decrease in the hospitalizations due to injuries, which have become the third cause of hospitalization, and a simultaneous increase in the hospitalizations for reasons related to maternity, and neoplasms. The need for healthcare increased together with an influx of elderly people as well as those suffering from chronic diseases whose treatment was difficult or even stopped due to the war outbreak [5, 10, 25]. On the other hand, for paediatric patients three main causes of hospitalizations changed completely, and the infectious diseases from the ninth place in the ranking got into the first place.

The epidemiological situation in the country of origin is an important factor when considering diseases in newly arrived migrants and refugees [26, 27]. Ukraine shows the highest global burdens of noncommunicable diseases (including ischemic heart disease, stroke, diabetes, cirrhosis, lung cancer, cardiomyopathy and alcohol use disorders) and chronic infectious diseases, such as tuberculosis, human immunodeficiency virus (HIV) and viral hepatitis, and the lowest vaccination coverage in Europe [18, 28, 29].

Infectious diseases often threaten the health of migrant populations and host communities. Well-documented outbreaks of vaccine-preventable diseases among migrants and refugees, children in particular, such as measles, rubella or COVID-19 have been reported [30–34]. Regarding the observed increase in the COVID-19 related hospitalizations, it is worth noticing that vaccinations against COVID-19 in Ukraine started in February 2021, and vaccination coverage is very low (only 36.93% of the population received two doses) [35]. It is of key importance to provide migrants in the host countries with access to vaccinations [18, 36].

The finding of the wider spread of infectious diseases among refugees, especially among children and adolescents, is consistent with the previous analyses of migration data and may be partially attributable to vaccination coverage, pre-migration experiences, including poor sanitation and nutrition, reduced healthcare access that could be associated with an increased risk of hospitalization after refugees’ arrival in a host country [37, 38].

There is clear evidence of a high demand for medical services among Ukrainian women for reasons associated with maternal health. Studies by other authors show that migrant women from the conflict-zone countries developed an increased risk of adverse health and mental health outcomes as well as neonatal mortality and morbidity [39–41]. Providing optimal perinatal care will result in reducing health inequalities in this vulnerable group, both refugee women and their babies [41].

Neoplasms are among main causes of hospital discharges among migrants. This highlights the importance of analysing this pathology in migrant populations. It has been reported that the situation related to oncological treatment in Ukraine is difficult, and there is a need for global support in this respect to ease the burden for Ukrainian patients [25, 42–46].

There are limitations to this study. First, considering the lack of precise data on the number of Ukrainian citizens residing in Poland (in the critical period, this number considerably changed every day and the influx to Poland was accompanied by the outflux to Ukraine and third countries), the authors decided to compare the structure of hospitalizations of Ukrainian citizens before and after 24.02.2022, and to put stress on the changing needs of the migrants instead of on the epidemiological situation. Second, information on the causes and frequency of hospitalizations clearly does not provide a complete picture of the health situation of migrants and war refugees because hospitalization depends on the severity of the disease, the possibility of establishing a diagnosis and providing proper treatment outside hospital, availability of hospital beds, and socio-economic factors. This study does not capture other aspects of health system being used by migrants and refugees (e.g., primary care) that may relate to health conditions that do not require hospitalization. Data from hospital discharge records usually represent severe outcomes of the spectrum of diseases and health problems, so it would be worth interpreting them in the context of other indicators that describe migrants’ health. Third, the years 2020–2021 were not typical regarding the hospitalized morbidity in Poland due to changes in the functioning of hospitals related to the COVID-19 pandemic. However, considering that these limitations apply to both the pre-war migrant cohort and war refugees, this study offers an unique opportunity to compare hospitalizations between these populations to identify healthcare use patterns and health trends related to war migration. More research needs to be done to better understand how to deliver healthcare services to migrants and war refugees.

## Conclusions and further research

This study generates new knowledge on hospitalization events among Ukrainian migrants and war refugees. It presents changes in the frequency of hospitalizations, leading causes of hospital admissions and age structure of the population using hospital services after February 24, 2022.

In the case of a sudden influx of a large number of war refugees to a host country, any information on the frequency and structure of hospitalized morbidity is extremely valuable for fulfilling the unmet health needs of war refugees, alleviating the burdens in the health system, developing new healthcare solutions and planning the health policy of the state for the next months and years.

Health system in the host country must be ready to provide services to pregnant women, children with infectious diseases as well as people with chronic diseases and underlying conditions that require the continuity of care.

Providing adequate standard of healthcare for refugees and migrants is important to the health of the entire population and is essential for the protection of the human rights of refugees, migrants and communities of host countries.

## Data Availability

The datasets supporting the conclusions of this article are available in the National Institute of Public Health NIH – National Research Institute (NIPH NIH-NRI) repository and can be made available to researchers in accordance with the legal restrictions. This dataset is created as a part of the Nationwide General Hospital Morbidity Study (NGHMS), within the framework of the Programme of Statistical Surveys of Official Statistics in Poland. Researchers can apply for access to the information that is available in the public administrative registries in Poland such as NGHMS. The application must contain specifications of the data requested and determination of the research purposes. To request access to this data contact NIPH NIH-NRI at: pzh@pzh.gov.pl.

## Abbreviations

WHO: World Health Organization
NGHMS: Nationwide General Hospital Morbidity Study
ICD-10: International Statistical Classifications of Diseases and Related Health Problems, 10th Revision
COVID-19: Coronavirus Disease 2019
HIE: Healthy immigrant effect
HIV: Human immunodeficiency virus
